# Environmentally Relevant Concentrations of Tetracycline Promote Horizontal Transfer of Antimicrobial Resistance Genes via Plasmid-Mediated Conjugation

**DOI:** 10.3390/foods13111787

**Published:** 2024-06-06

**Authors:** Haibo Zhou, Zhaoxin Lu, Xinmei Liu, Xiaomei Bie, Feng Xue, Sijie Tang, Qiushi Feng, Yiyu Cheng, Jun Yang

**Affiliations:** 1 College of Food Science and Technology, Nanjing Agricultural University, Nanjing 210095, China; 2020208002@stu.njau.edu.cn (H.Z.);; 2 Key Laboratory of Detection and Traceability Technology of Foodborne Pathogenic Bacteria for Jiangsu Province Market Regulation, Nanjing Institute for Food and Drug Control, Nanjing 211198, China; 3 MOE Joint International Research Laboratory of Animal Health and Food Safety, Nanjing Agricultural University, Nanjing 210095, China

**Keywords:** tetracycline, conjugative transfer, antimicrobial resistance genes, reactive oxygen species, cell membrane permeability

## Abstract

The ubiquitous presence of antimicrobial-resistant organisms and antimicrobial resistance genes (ARGs) constitutes a major threat to global public safety. Tetracycline (TET) is a common antimicrobial agent that inhibits bacterial growth and is frequently detected in aquatic environments. Although TET may display coselection for resistance, limited knowledge is available on whether and how it might influence plasmid-mediated conjugation. Subinhibitory concentrations (3.9–250 ng/mL) of TET promoted horizontal gene transfer (HGT) via the mobilizable plasmid pVP52-1 from the donor *Vibrio parahaemolyticus* NJIFDCVp52 to the recipient *Escherichia coli* EC600 by 1.47- to 3.19-fold. The transcription levels of tetracycline resistance genes [*tetA*, *tetR*(A)], conjugation-related genes (*traA*, *traD*), outer membrane protein genes (*ompA*, *ompK*, *ompV*), reactive oxygen species (ROS)-related genes (*oxyR*, *rpoS*), autoinducer-2 (AI-2) synthesis gene (*luxS*), and SOS-related genes (*lexA, recA*) in the donor and recipient were significantly increased. Furthermore, the overproduced intracellular ROS generation and increased cell membrane permeability under TET exposure stimulated the conjugative transfer of ARGs. Overall, this study provides important insights into the contributions of TET to the spread of antimicrobial resistance.

## 1. Introduction

Antimicrobial resistance is one of the greatest global challenges to human and animal health. In fact, the misuse of drugs is the main risk factor leading to antimicrobial resistance [1,2]. If no powerful interventions are implemented in a timely manner, approximately 10 million people will die due to antimicrobial resistance every year by 2050 [3]. This resistance has two main pathways: vertical gene transfer (VGT) through errors in DNA replication and horizontal gene transfer (HGT) through the acquisition of antimicrobial resistance genes (ARGs) between bacteria [4]. Among the three primary pathways of HGT (i.e., transformation, conjugation, and transduction), conjugation involving a pilus bridge between bacterial cells is considered particularly important for the transmission of ARGs [5]. Over the past few decades, to meet the growing demand for animal protein production, the increasing application of antimicrobial agents has become necessary in low- and middle-income countries [2,6]. In China, tetracycline (TET) is typically used as a veterinary drug for disease prevention, disease treatment, or growth promotion in the fields of livestock and aquaculture, resulting in the presence of its residuals in aquatic environments at concentrations ranging from ng/L to μg/L [7,8].

It is generally assumed that antimicrobial agents are the major drivers of antimicrobial resistance and produce selective pressure to enhance the adaptability of bacteria carrying mobile genetic elements (MGEs), such as resistance plasmids, in environmental settings [9,10]. Previous studies have demonstrated that some antimicrobial agents (quinolones) can facilitate the development of antimicrobial resistance via mutation and transformation [11,12]. Currently, many nonantibiotic substances, such as heavy metals [13], antidepressants [14], and nanoparticles [15], have also been shown as facilitating the conjugative transfer of ARGs. However, little is known about the underlying mechanisms involved in the TET-triggered horizontal transfer of plasmid-mediated ARGs from *Vibrio parahaemolyticus*. *V. parahaemolyticus* is an important zoonotic pathogen with high morbidity rates worldwide, predominantly causing gastroenteritis and diarrhea, which are frequently caused by the ingestion of undercooked or raw seafood [16,17]. In addition, our recent study reported that *V. parahaemolyticus* isolates from retail aquatic products exhibited a high level of resistance to TET [18].

Therefore, we selected TET to investigate whether and how it influenced the conjugative transfer of ARGs at environmentally relevant concentrations (from 3.9 ng/mL to 250 ng/mL). In this study, a conjugative mating system was established with a mobilizable plasmid to test the transfer frequency post-TET exposure. In the present work, we systematically studied the impacts of TET on the conjugative transfer of ARGs from *V. parahaemolyticus* NJIFDCVp52 (donor) to *E. coli* EC600 (recipient) and the underlying mechanisms involved. The NJIFDCVp52 strain carries the IncA/C_2_ multidrug-resistant plasmid pVP52-1, which was isolated from an aquatic product in a local seafood market (Nanjing, China). Comprehensive studies involving phenotypic and genotypic analyses, including culture-based conjugation experiments, reactive oxygen species (ROS) generation, cell membrane permeability, and expression levels of related genes, were conducted. These findings provide evidence regarding the role of TET in the conjugation-induced transmission of antibiotic resistance in aquatic environments.

## 2. Materials and Methods

### 2.1. Bacterial Strains and Reagents

The donor *V. parahaemolyticus* NJIFDCVp52 strain was obtained from our previous study [18] and harbored the conjugative plasmid pVP52-1 (172,213 bp), which carries genes conferring resistance to cefotaxime (CTX), chloramphenicol (CHL), tetracycline (TET), and trimethoprim/sulfamethoxazole (SXT). The recipient *E. coli* EC600 strain was resistant to rifampicin (RIF). The two bacterial strains were preserved by the Microbiology Laboratory, Nanjing Institute for Food and Drug Control. All antimicrobials were purchased from Aladdin Co., Ltd. (Shanghai, China). Antimicrobial disks containing ampicillin (AMP), nalidixic acid (NAL), ampicillin/sulbactam (SAM), ceftazidime (CAZ), cefoxitin (FOX), cefazolin (CFZ), imipenem (IPM), azithromycin (AZI), CTX, CHL, TET, and SXT were obtained from Hangzhou Microbial Reagent Co., Ltd. (Hangzhou, China).

### 2.2. Antimicrobial Susceptibility Testing

The antimicrobial resistance profiles of the donor (*V. parahaemolyticus* NJIFDCVp52), the recipient (*E. coli* EC600), and the transconjugant (*E. coli* Vp52-EC600) against 12 antimicrobial agents were determined by the Kirby–Bauer disk diffusion method. The minimum inhibitory concentrations (MICs) of TET for all strains were further confirmed by the microdilution broth method. The results were interpreted according to the Clinical and Laboratory Standards Institute (CLSI) guidelines [19,20]. The detailed experimental steps were performed with reference to the method described previously [11,18].

### 2.3. Bacterial Growth Inhibition

The inhibitory effect of TET on the growth of the donor strain (NJIFDCVp52) and recipient strain (EC600) was examined by the plate count method. In brief, NJIFDCVp52 and EC600 were grown in Luria–Bertani broth (LB, Beijing Land Bridge, Beijing, China) and incubated to the logarithmic growth stage at 37 °C with shaking. Then, each bacterial culture was adjusted to a 0.5 McFarland turbidity standard, which was exposed to different concentrations of TET (3.9, 7.8, 15.6, 31.3, 62.5, 125, 250, and 500 ng/mL). Treatments excluding drugs were used as negative controls. After 4 h of incubation at 37 °C, the fresh cultures were washed three times and then serially diluted with 0.85% (*w*/*v*) sterilized NaCl solution (sterile saline), and 100 μL of bacterial suspension was plated. Three independent experiments were performed with five replicates.

### 2.4. Conjugation Experiments

The conjugation assay followed the method described in previous work [21] with minor modifications. Briefly, both the donor and recipient strains were cultured to the logarithmic growth stage in LB broth. Overnight cultures were streaked onto Eosin–Methylene blue agar (EMB, Beijing Land Bridge) medium supplemented with either 4 μg/mL TET, 64 μg/mL RIF, or both antimicrobials (4 μg/mL TET and 64 μg/mL RIF); the cultures were then incubated at 37 °C for 24–48 h and checked for microbial growth. Moreover, the overnight cultures were collected by centrifugation and resuspended in a 0.5 McFarland turbidity standard in 0.85% (*w*/*v*) sterilized NaCl solution (sterile saline). Two hundred microliters of the donor strain (NJIFDCVp52) and 200 μL of the recipient strain (*E. coli* EC600) were thoroughly mixed at a ratio of 1:1. Diluted drugs at different concentrations (0, 3.9, 7.8, 15.6, 31.3, 62.5, 125, and 250 ng/mL) were added to the mating systems and statically incubated at 37 °C for 4 h. The conjugation mixture was then washed three times, diluted with sterile saline, and uniformly plated onto EMB agar selection plates. Transconjugants were identified by PCR detection of resistance genes located on transferable plasmids. The frequency was calculated as the number of transconjugants (EMB plates containing 4 μg/mL TET and 64 μg/mL RIF) divided by the number of recipient cells (EMB plates containing 64 μg/mL RIF) [22]. The values were expressed as the mean ± standard deviation of three independent experiments.

### 2.5. Determination of ROS Generation

The intracellular ROS production of the donor strain NJIFDCVp52 and recipient strain EC600 was measured using 2′,7′-dichlorodihydrofluorescein diacetate (DCFH-DA) (Solarbio, Beijing, China), following the manufacturer’s protocols. The bacterial cells in the logarithmic growth period were washed twice with PBS (pH 7.4, Solarbio) and resuspended to approximately 10^6^ CFU/mL. Bacterial suspensions were incubated individually with DCFH-DA (at a final concentration of 10 μmol/L) at 37 °C for 30 min in the dark. During this period, the mixture was gently shaken every 5 min to allow full contact between the dye and cells. After incubation, the tubes were washed with PBS three times to fully remove the unbound dye. Then, TET at different concentrations (0, 3.9, 7.8, 15.6, 31.3, 62.5, 125, and 250 ng/mL) was added to the bacterial solutions for 4 h at 37 °C in the dark. The fluorescence intensity (excitation wavelength = 488 nm; emission wavelength = 525 nm) of each sample was detected in a 96-well plate (Corning, NY, USA) at 200 μL per well using a SpectraMax M3 reader (Molecular Devices, Silicon Valley, CA, USA). Independent experiments were performed in triplicate under each condition.

### 2.6. Cell Membrane Permeability Assay

The effects of TET on outer membrane permeability were measured using N-phenyl-1-naphthylamine (NPN). Logarithmic phase bacterial cultures were washed twice and adjusted to approximately 10^6^ CFU/mL in PBS (pH 7.4). Various amounts of TET were added to final doses of 0 (negative control), 3.9, 7.8, 15.6, 31.3, 62.5, 125, and 250 ng/mL, and then incubated at 37 °C for 4 h. After washing three times, NPN was added at a final concentration of 10 μmol/L at 37 °C for 30 min in the dark. To further evaluate the effect of TET on inner membrane permeability, propidium iodide (PI) (Solarbio) was applied. The fluorescence intensities of NPN and PI were determined by a microplate reader (Molecular Devices). The measurement parameters were as follows: NPN, excitation wavelength of 350 nm and emission wavelength of 420 nm; PI, excitation wavelength of 535 nm and emission wavelength of 615 nm.

### 2.7. RNA Extraction and RT-qPCR

Bacterial cells were treated with TET at 0 (negative control), 3.9, 7.8, 15.6, 31.3, 62.5, 125, and 250 ng/mL for 4 h at 37 °C and centrifuged at 6000× *g* for 10 min. Total RNA was extracted using a FastPure^®^ Cell/Tissue Total RNA Isolation Kit (Vazyme, Nanjing, China). Residual DNA contamination was removed by gDNA-Filter Columns. Then, the RNA was immediately reverse-transcribed into cDNA using HiScript^®^ II qRT SuperMix (Vazyme). The expression of TET resistance genes [*tetA*, *tetR*(A)], conjugation-related genes (*traA*, *traD*), outer membrane protein genes (*ompA*, *ompK*, *ompV*, *ompW*, *tolB*, *tolC1*, *tolC2*), ROS-related genes (*oxyR*, *rpoS*), autoinducer-2 (AI-2) synthesis genes (*luxS*), and SOS-related genes (*lexA, recA*) from the donor strain NJIFDCVp52 was quantified by quantitative PCR (qPCR) analysis. Additionally, the expression of outer membrane protein genes (*ompA*, *ompF*), ROS-related genes (*oxyR*, *rpoS*), AI-2 synthesis genes (*luxS*), and SOS-related genes (*lexA, recA*) from the recipient strain EC600 was also assessed. The 16S rRNA [23] was used as an internal reference gene to normalize the gene expression level based on the 2^−ΔΔCt^ method. The primers used for qPCR are detailed in Table S1. All qPCR assays were performed in triplicate using ChamQ SYBR Master Mix (Vazyme) in a C1000 Touch^TM^ Thermal Cycler (Bio-Rad, Hercules, CA, USA).

### 2.8. Statistical Analysis

Statistical analysis was implemented using GraphPad Prism 8.0.1 software (GraphPad Software Inc., Solana Beach, CA, USA). The significance of differences between the control and treatment groups was evaluated using one-way analysis of variance (ANOVA). Differences were considered to be statistically significant at *p* values less than 0.05. All experiments were carried out in biological triplicates.

## 3. Results

### 3.1. Bacterial Growth Inhibition by TET

**Figure 1:**
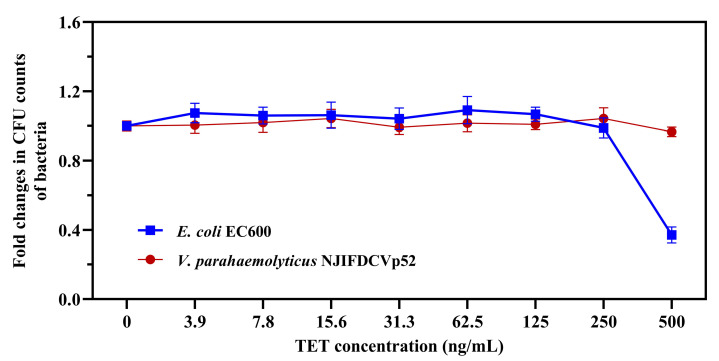
Tetracycline dose-dependent growth inhibition curves of *V. parahaemolyticus* NJIFDCVp52 and *E. coli* EC600.

The MICs of TET against the donor strain (*V. parahaemolyticus* NJIFDCVp52) and the recipient strain (*E. coli* EC600) were 8 μg/mL and 2 μg/mL, respectively (Table 1). First, the effect of subinhibitory TET on bacterial growth was studied based on the MICs measured in the present study. As demonstrated in Figure 1, the growth of *V. parahaemolyticus* NJIFDCVp52 and *E. coli* EC600 was not significantly affected by the dose of TET in the range of 3.9–250 ng/mL compared with that of the control (without TET treatment). However, the higher concentration (500 ng/mL) significantly inhibited the growth of *E. coli* EC600, with an inhibition rate higher than 60%. Therefore, 3.9–250 ng/mL TET was selected for subsequent experiments.

### 3.2. Effects of TET on Plasmid Conjugative Transfer

**Figure 2:**
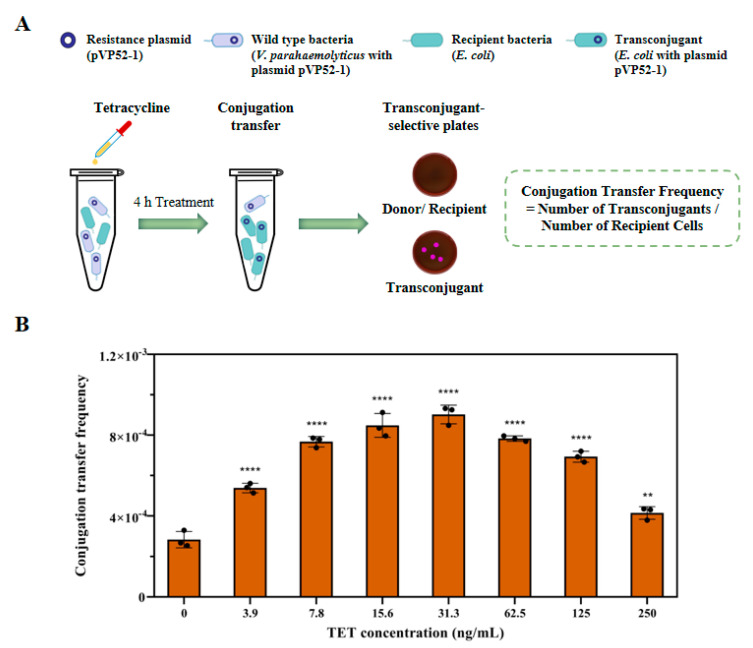
Conjugative transfer of the pVP52-1 plasmid from *V. parahaemolyticus* NJIFDCVp52 to *E. coli* EC600 induced by different concentrations of tetracycline. (**A**) Schematic of the conjugation assay; (**B**) effects of tetracycline on the conjugative transfer of the plasmid pVP52-1. Asterisks indicate significant difference (** *p* < 0.01, **** *p* < 0.0001).

To investigate the effects of TET on plasmid conjugative transfer, we conducted conjugation assays under exposure to different sub-MIC concentrations of TET (in the range of 3.9–250 ng/mL). The experimental design and procedure are displayed in Figure 2A. In the presence of TET, the conjugation transfer frequency from *V. parahaemolyticus* NJIFDCVp52 to *E. coli* EC600 significantly increased (*p* < 0.05) after 4 h of mating and peaked at 31.3 ng/mL TET, showing a trend of first increasing and then decreasing (Figure 2B). The transfer frequency was determined to be 2.83 × 10^−4^ without TET exposure. In particular, the conjugation transfer frequency of the plasmid (ranging from 4.15 × 10^−4^ to 9.02 × 10^−4^) increased by approximately 1.47–3.19-fold compared with that of the control. Overall, these results confirmed that TET considerably improved the conjugative transfer of multiple ARGs mediated by the mobilizable plasmid at environmentally relevant concentrations.

### 3.3. Effects of TET on ROS Generation

**Figure 3:**
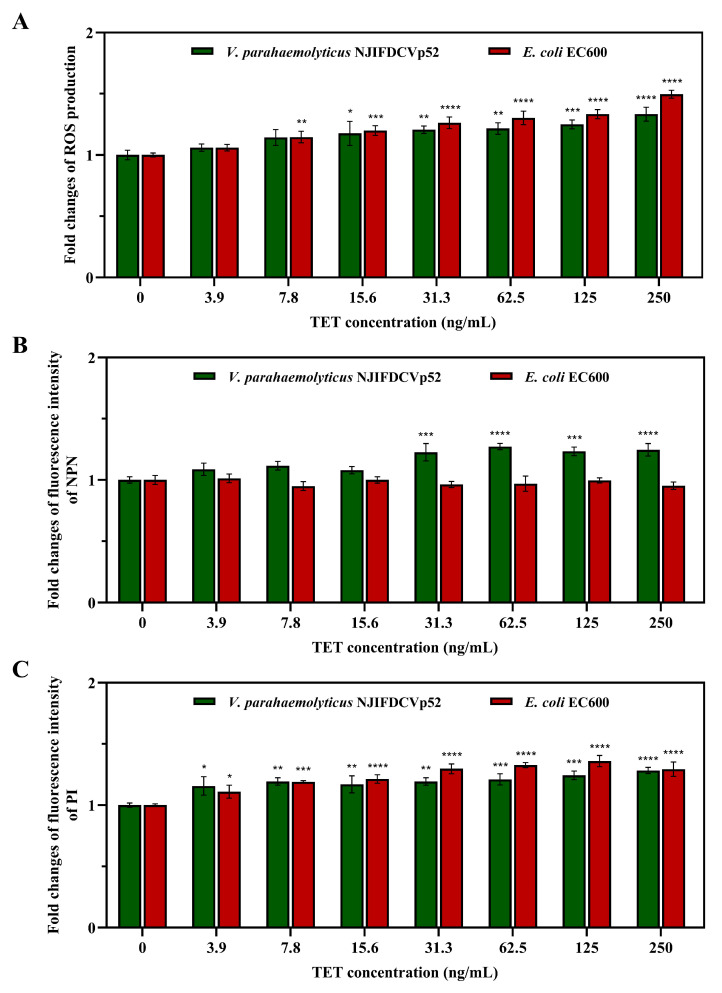
Effects of tetracycline on ROS generation and membrane permeability in the donor (*V. parahaemolyticus* NJIFDCVp52) and recipient (*E. coli* EC600) strains. (**A**) Fold changes in ROS production; (**B**) fold changes in the fluorescence intensity of NPN; (**C**) fold changes in the fluorescence intensity of PI. Asterisks indicate significant difference (* *p* < 0.05, ** *p* < 0.01, *** *p* < 0.001, **** *p* < 0.0001).

To examine the effects of TET on ROS generation in donor and recipient bacteria, intracellular ROS levels were determined by DCFH-DA dye after exposure to various concentrations of TET. DCFH-DA has been extensively used to detect ROS because it can freely pass through the cell membrane, where it is hydrolyzed into DCFH with poor permeability. DCFH can then be oxidized by ROS to generate fluorescent DCF that can be detected. Here, ROS production within the donor and strain recipient increased with increasing concentrations of TET (Figure 3A). Interestingly, the stimulatory effect of TET at concentrations between 7.8 ng/mL and 250 ng/mL on the recipient strain EC600 (1.06–1.50-fold) was more obvious than that on the donor strain NJIFDCVp52 (1.06–1.33-fold). In contrast, a low concentration of TET (3.9 ng/mL) had no effect on ROS generation compared with the control without TET exposure. As a positive control, the Rosup reagent (100 μg/mL) can cause significant ROS generation in both the donor (1.89-fold) and recipient (2.61-fold) compared to the corresponding negative control.

### 3.4. Effects of TET on Cell Membrane Permeability

In general, the cell membrane structure of Gram-negative bacteria is composed of an outer membrane (OM) and an inner membrane (IM). The changes in permeability of both the donor *V. parahaemolyticus* NJIFDCVp52 and recipient *E. coli* EC600 strains were evaluated using NPN and PI dye, respectively. First, NPN was used to detect the permeability of OM. NPN is usually unable to cross undamaged OM and displays weak fluorescence in aqueous solution; however, it shows increased fluorescence only when entering hydrophobic environments through damaged OM. Figure 3B shows that the addition of TET at concentrations ranging from 31.3 ng/mL to 250 ng/mL led to a significant increase (*p* < 0.001) in the NPN fluorescence of the donor strain. Conversely, within the tested concentration range of TET, the NPN fluorescence of the recipient strain did not significantly change (*p* > 0.05), indicating that TET did not enhance the permeability of the OM. Furthermore, the PI was applied to evaluate the permeability of the IM. PI is a DNA stain reagent that enters compromised cell membranes, emitting red fluorescence. As shown in Figure 3C, the IM permeability of the donor and recipient strains was significantly enhanced at the tested TET concentrations in this study. In comparison, the IM permeability of *E. coli* EC600 (1.11- to 1.36-fold) was more susceptible to TET treatment than that of *V. parahaemolyticus* NJIFDCVp52 (1.16- to 1.28-fold), which is similar to ROS generation. Heat-killed bacteria were used as positive controls, significant enhancements of the NPN fluorescence were observed in both the donor (3.77-fold) and recipient (4.86-fold) compared to the negative control group, while the same trend in the PI fluorescence was observed in both the donor (3.91-fold) and recipient (5.24-fold).

### 3.5. Gene Expression under Subinhibitory TETs

To further elucidate the underlying mechanism of enhanced conjugative transfer from a genetic perspective, the transcription levels of important genes were evaluated in response to TET exposure.

#### 3.5.1. Differential Expression of Genes in the Donor Strain

**Figure 4:**
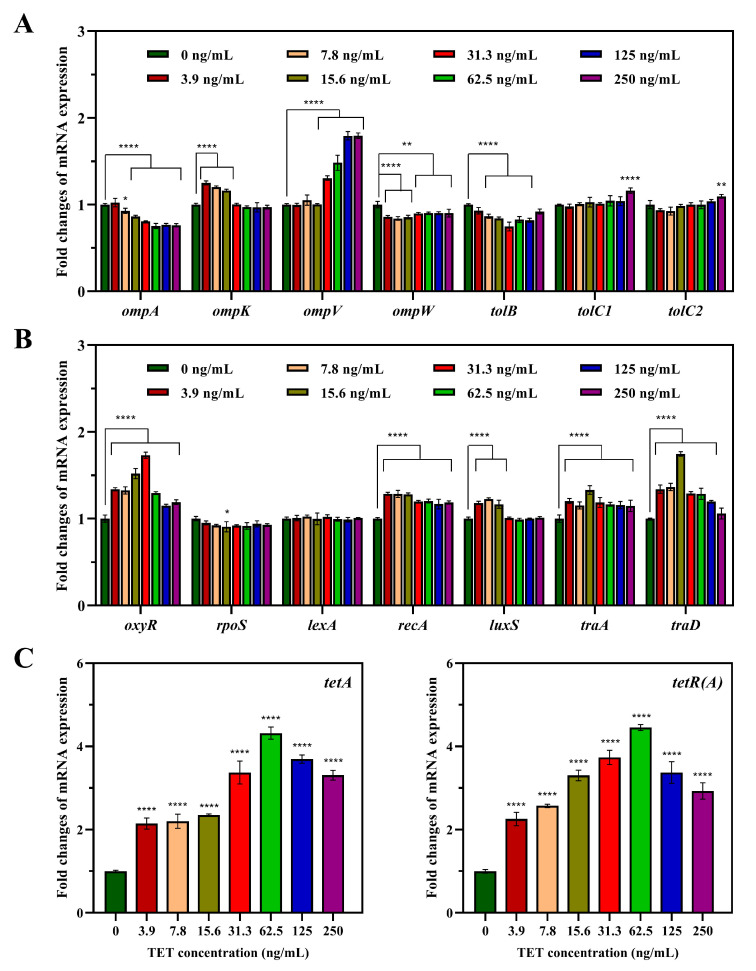
Effect of tetracycline on the mRNA expression levels of NJIFDCVp52-related genes. (**A**) Fold changes in the expression of genes related to outer membrane proteins in the presence of different concentrations of TET relative to those in the presence of a drug-free solvent; (**B**) fold changes in the expression of genes related to ROS production, the SOS response and conjugation; (**C**) fold changes in the expression of genes related to tetracycline resistance. Asterisks indicate significant difference (* *p* < 0.05, ** *p* < 0.01, **** *p* < 0.0001).

The outer membrane proteins (OMPs) of Gram-negative bacteria determine the permeability of the membrane barrier, which plays critical roles in the exchange of material between the cell and the external environment. In the donor *V. parahaemolyticus* NJIFDCVp52 (Figure 4A), the mRNA expression of *ompK* and *ompV* was significantly increased by 0.97–1.25-fold and 1.00–1.80-fold, respectively. Nevertheless, TET significantly decreased the expression levels of *ompA*, *ompW*, and *tolB* by 0.75–1.02-fold, 0.84–0.90-fold, and 0.75–0.93-fold, respectively. The expression levels of *tolC1* and *tolC2* showed no obvious changes, with the exception of when at a high concentration (250 ng/mL).

In addition, as shown in Figure 4B, the levels of the ROS regulatory gene *oxyR* and the SOS response-related gene *recA* increased by approximately 1.15- to 1.73-fold and 1.17- to 1.29-fold, respectively. The AI-2 synthesis-related gene *luxS* showed an increase of 1.17–1.22-fold under exposure to 3.9–15.6 ng/mL TET. Conjugative transfer-related genes (*traA* and *traD*) were upregulated by 1.15–1.33-fold and 1.06–1.75-fold, respectively. The expression of tetracycline resistance genes [*tetA*, *tetR(A)*] increased 2- to 5-fold, peaking at 62.5 ng/mL TET (Figure 4C).

#### 3.5.2. Differential Expression of Genes in the Recipient Strain

**Figure 5:**
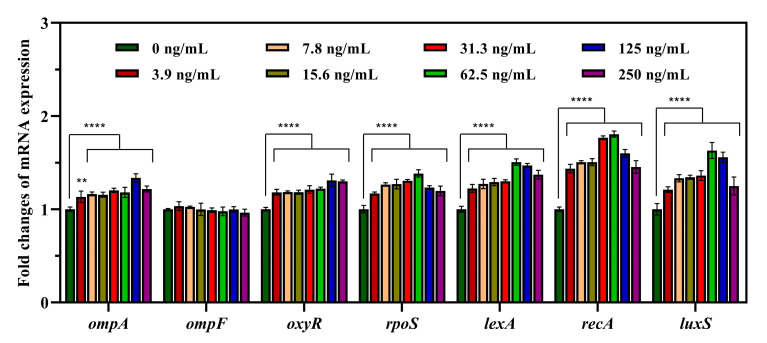
Effect of tetracycline on the mRNA expression levels of EC600-related genes. Asterisks indicate significant difference (** *p* < 0.01, **** *p* < 0.0001).

In the recipient *E. coli* EC600, the expression of the *ompA* gene, which encodes a representative OMP, was significantly upregulated, with fold changes ranging from 1.13 to 1.34 (Figure 5), while the expression of *ompF* showed no obvious changes under TET stress. In addition to OMP-related genes, multiple other genes related to ROS production (*oxyR*, *rpoS*), the SOS response (*lexA*, *recA*), and AI-2 synthesis (*luxS*) were also upregulated to various degrees. Specifically, the transcription level of *oxyR* gradually increased as the TET concentration increased, with up to 1.31-fold upregulation detected. The expression of *rpoS, lexA, recA*, and *luxS* increased by 1.17–1.38-fold, 1.22–1.51-fold, 1.44–1.81-fold, and 1.21–1.63-fold, respectively. Similarly, the expression of *rpoS, lexA, recA*, and *luxS* increased, first increasing but then decreasing, with the greatest increase detected at 62.5 ng/mL TET.

## 4. Discussion

**Figure 6:**
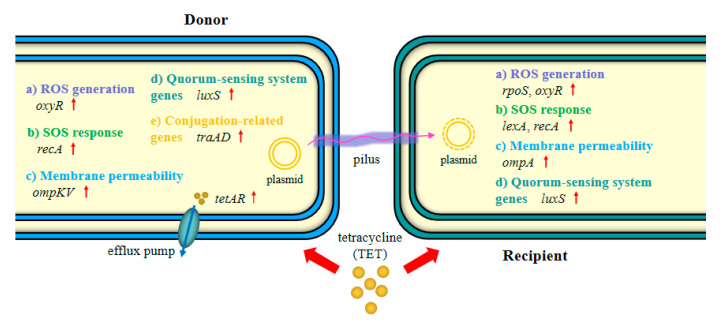
Schematic of the underlying mechanisms by which tetracycline promoted the conjugative transfer of plasmid-borne ARGs. Tetracycline can promote conjugation via (1) induced overproduction of ROS, (2) stimulated the SOS response, (3) increased cell membrane permeability, and (4) stimulated expression of conjugation-related genes. Red arrows indicate upregulated expression.

The emergence and spread of antimicrobial resistance in bacteria is a substantial challenge to microbial ecology, public health, and the economy worldwide. TET is one of the most commonly used antimicrobial agents to prevent and treat infectious diseases in agricultural, veterinary, and clinical settings [7,24]. In addition, according to the National Food Safety Standard for Maximum Residue Limits (MRLs) for veterinary drugs in foods [25], TET can be detected with relatively high MRLs (100–1200 μg/kg) in livestock meat, poultry meat, and fish. Although some previous reports have indicated that antimicrobial agents can cause coselection and resistance [26,27,28], the mechanism underlying the effects of antimicrobial agents on plasmid-mediated ARG transfer remains unclear. In the present work, our phenotypic conjugation experiments showed that TET at environmentally relevant concentrations (3.9–250 ng/mL) could obviously enhance the transfer frequency of ARGs and reached a maximum at 31.3 ng/mL TET. Jutkina et al. [29] reported that TET mostly promoted HGT from the effluent bacterial community to the recipient strain *E. coli* CV601 at 10 ng/mL, an increase of 4 times, which is generally consistent with the findings of this study. To elucidate the underlying mechanisms by which TET promoted the conjugative transfer of ARGs from the *V. parahaemolyticus* strain to the *E. coli* strain, multiple methods were employed in this study, including determination of ROS generation, cell membrane permeability tests, and quantitative transcript analysis. Taken together, the main mechanisms proposed, including the overproduction of ROS, stimulation of the SOS response, increased cell membrane permeability, and upregulation of the expression of conjugation-related genes, might play important roles in the enhanced conjugation of multidrug resistance plasmids (Figure 6).

Recent reports have revealed that various environmental pollutants can induce the overproduction of ROS [13,14,15]; ultimately, this can affect the conjugative transfer frequency of mobilizable plasmids. Our findings showed that ROS production levels increased significantly with increasing TET concentrations, especially in the recipients (a 1.06- to 1.50-fold increase). In addition, the expression of the ROS regulatory genes *oxyR* and *rpoS* under TET exposure was evaluated. The expression of *oxyR* in *V. parahaemolyticus* NJIFDCVp52 (donor) and the expression of *rpoS* in *E. coli* EC600 (recipient) first increased and then decreased, roughly consistent with the dynamic trends of conjugative transfer frequency. The sigma factor RpoS and the ROS-sensing transcriptional regulator OxyR are typically expressed under particular stress conditions [30,31]. Moreover, to decrease the damage caused by increased intracellular ROS production, both donor and recipient strains may activate the DNA repair process of the SOS system [32,33]. The RecA (inducer) and LexA (repressor) are important proteins involved in the SOS response and serve as primary comeback survival pathways for organisms [34]. Indeed, the expression of *recA* in the donor and the expression levels of *lexA* and *recA* in the recipient were upregulated following treatment with TET. Similarly, antidepressants and dimethyl phthalate could also enhance the expression of *lexA* and *recA* [14,35]. Thus, suitable ROS levels play an important role in ARG transfer, and the increased expression of genes associated with ROS and SOS responses is involved in conjugative transfer under stress.

The bacterial cell membrane serves as an important barrier that regulates the horizontal transfer of DNA material among bacteria, including plasmid conjugation. Therefore, the permeability of the outer membrane and inner membrane was examined using NPN and PI tests, respectively. Interestingly, the outer membrane permeability of *V. parahaemolyticus* NJIFDCVp52 was more sensitive to TET treatment than that of *E. coli* EC600, while the opposite trend was observed for inner membrane permeability. This may be due to species-specific differences. IM permeability experiments showed that PI can readily enter cells with damaged membranes and emit fluorescence, which further confirms the destructive effect of TET on bacterial cell membranes. Furthermore, we speculated that the increased expression of inner membrane proteins might assist the entering of foreign plasmid DNA to bacterial cytoplasm. However, this hypothesis will require further experimental validation. Therefore, it is necessary to carry out in-depth research on expression levels of IM-related proteins and protein–protein interactions. Cell membrane permeability is generally associated with outer membrane proteins, which mainly consist of lipopolysaccharides (LPS), phospholipids (PLs), and porins [36,37]. In particular, several porin proteins, including OmpA, OmpC, OmpF, OmpK, OmpV, OmpW, and efflux pumps (TolB, TolC), are known to play vital roles in the membrane permeability of donor and recipient bacteria. For the donor strain, increased transcription levels of relevant genes coding for outer membrane proteins were detected under TET exposure, while the gene *ompA* was downregulated. OmpK and OmpV are the major outer membrane proteins among *Vibrio* spp., which are highly immunogenic, and are closely related to environmental adaptation. The observed changes in the mRNA expression levels of *ompK* and *ompV* might indicate that TET has an effect on the function of these outer membrane proteins, which creates favorable conditions for plasmid transfer. In the future, we will conduct gene knockout experiments to clarify the relevant molecular mechanisms. In contrast, the mRNA expression of *ompA* was upregulated in the recipient strain. It is suggested that the enhanced expression of membrane protein-coding genes plays a crucial role in alterations of membrane permeability. Previous studies have reported that ROS overproduction may cause cell damage in either donor or recipient bacteria and promote conjugative transfer [14,15,38].

To further investigate other factors contributing to conjugation, the expression of genes related to the conjugative transfer process and the quorum-sensing (QS) system was quantified in our study. Direct cell-to-cell connections are a prerequisite for the conjugative transfer of plasmids between individual bacterial cells. In this study, transcriptional analyses suggested that the expression of conjugative transfer protein-encoding genes (*traA* and *traD*) increased in response to exposure to TET, and this increase became more obvious with decreasing TET dosage. *traA* (384 bp) is a group of *tra* genes encoding the type IV secretion system (T4SS) that forms the mating pair formation (Mpf) system on the broad-host-range plasmid pVP52-1. The gene *traD* (1863 bp), encoding the type IV coupling protein (T4CP), belongs to the DNA transfer and replication (Dtr) system. Unlike that in the donor strain, the expression of the AI-2 synthesis gene *luxS* was enhanced in the recipient strain at both low and high TET concentrations. QS is a bacterial intercellular communication system through secreted signaling molecules such as AI-2, which regulate various physiological activities in response to changes in the external environment [39,40]. Overall, conjugative transfer is influenced by the states of both the donor and recipient strains rather than solely relying on one side.

Notably, plasmids are among the most commonly identified types of exogenous DNA; they participate not only in conjugation between living bacteria but also as important cell-free DNA released from damaged or dead bacteria that promotes the exchange of ARGs via the transformation process in competent bacteria [41]. In our study, the plasmid pVP52-1 was effectively transferred into the recipient (*E. coli* EC600), and the phenotypic resistance profile dramatically changed from AMP-NAL to AMP-SAM-CFZ-FOX-CAZ-CTX-IPM-NAL-CHL-TET-SXT-AZI (Table 1), which further indicates that plasmids carrying multiple ARGs are the primary route for the spread of antimicrobial resistance. For example, the significantly increased transcription of genes [*tetA*, *tetR*(A)] explains the molecular mechanism of TET resistance. *tetA* is a gene encoding the TetA efflux pump that can actively pump out drugs, conferring TET resistance. The *tetR*(A) gene encodes the transcriptional repressor protein TetR(A), which tightly regulates the expression of *tetA*. In the presence of TET, it binds to TetR(A), causing dissociation from the Tet Operator (TetO) sequence and subsequent restoration of *tetA* gene expression [42]. Taken together, our study systematically explored the mechanisms by which TET promotes the transfer of plasmid-mediated ARGs at environmentally relevant concentrations from a phenotypic and genetic perspective. Further studies are required to evaluate the effects of TET on the conjugative transfer of ARGs in other complex environments, e.g., aquatic products and aquaculture water, or in vivo animal models.

## 5. Conclusions

This study illustrated that TET could facilitate the conjugative transfer of ARGs mediated by the mobilizable plasmid pVP52-1 from the donor *V. parahaemolyticus* NJIFDCVp52 to the recipient *E. coli* EC600 at environmental concentrations. Enhanced intracellular ROS generation, increased cell membrane permeability, and upregulated expression of conjugation-related genes potentially contributed to the promotion of conjugative ARG transfer. Our findings suggest that TET residues should be carefully evaluated to mitigate the transmission risk of ARGs in various environments.

## Figures and Tables

**Table 1:** Resistance phenotypes of strains.

Antimicrobial Agents	Resistance Phenotype ^1^		
	NJIFDCVp52	EC600	Vp52-EC600
Ampicillin (AMP)	R	R	R
Ampicillin/sulbactam (SAM)	R	***S***	***R***
Cefazolin (CFZ)	R	***S***	***R***
Cefoxitin (FOX)	R	***S***	***R***
Ceftazidime (CAZ)	R	***S***	***R***
Cefotaxime (CTX)	R	***S***	***R***
Imipenem (IPM)	R	***S***	***R***
Nalidixic acid (NAL)	S	R	R
Chloramphenicol (CHL)	I	***S***	***R***
Tetracycline (TET)	I (8 μg/mL) ^2^	***S*** (2 μg/mL)	***R*** (16 μg/mL)
Trimethoprim/sulfamethoxazole (SXT)	R	***S***	***R***
Azithromycin (AZI)	S	***S***	***R***

^1^ R: resistant; I: intermediate; S: susceptible. Letters in bold italics indicate changes in the resistance phenotype before and after conjugation transfer. ^2^ Values in brackets are minimum inhibitory concentrations (MICs) of TET.

## Data Availability

The original contributions presented in the study are included in the article/Supplementary Materials, further inquiries can be directed to the corresponding authors.

## Supplementary Materials

The following supporting information can be downloaded at: https://www.mdpi.com/article/10.3390/foods13111787/s1, Table S1: Primers used for qPCR in this study.
