# Supplementary material for: Environmentally Relevant Concentrations of Tetracycline Promote Horizontal Transfer of Antimicrobial Resistance Genes via Plasmid-Mediated Conjugation
Source: Foods. 2024 Jun 6;13(11):1787. doi: 10.3390/foods13111787 (PMC11171790; doi:10.3390/foods13111787)
Supplement: Supplementary file 1 [file foods-13-01787-s001.zip › foods-3036528-supplementary.pdf]

Supplementary Materials

Table S1. Primers used for qPCR in this study.

Target gene	Forward primer (5’-3’)	Reverse primer (5’-3’)	Product size (bp)
**Donor strain**			
16S rRNA	TATCCTTGTTTGCCAGCGAG	CTACGACGCACTTTTTGGGA	186
tetA	CAGGCAGGTGGATGAGGAA	GCATAGATCGCCGTGAAGAG	105
tetR(A)	CCTCCTTTTCGCCAATCCA	CGTTGAACAGGCTCCGCTCT	124
traA	ATCCTGGTCGGTGTTGTTGG	CACTACGGTCGGGGAGTTGTA	102
traD	GCATTCGCTGATCGTTGG	CGCATCTCCTTATCCCCTTT	126
ompA	GGTGCGTACATGATGTCTGAAG	GCGTCCATATCGTCCCAAA	149
ompK	GACCCAGGCAGCGACAAA	GCCCCACTCCATTAGAGTAGAAAC	141
ompV	ATACTGCACCAATCCTAAACGG	GCTACATCGCCATCACGAATAA	154
ompW	AGGTGACTTCGTTCTTCGTGTT	CTCTAGGCTGATGTTGTCTGTGA	154
tolB	ATCGCCTTGCTCATTTTGC	GCAGTCAACCACTTCCTACCG	197
tolC1	TTAAGGCGTCTTGATCGTTTG	TCAGTGTAGCAGCACAGCGT	165
tolC2	GCCGATGCTGGTGGACTC	GCCTTTGACGGTTGGGTAG	145
oxyR	CTGCGGGAGCGGGTATTA	TGCGTGAAGGCGTAGGGT	105
rpoS	CTACTACCGAAGGCAAAACAGC	TCTGCGGGCGTAAAGGAC	140
lexA	CGAATGCCGCAGAGGAAC	GCAGCGACTTGACCAATCAG	145
recA	TGCTATGGCAAGACGAACTAGA	GTTTCAATACTTCCACATTTACGC	180
luxS	AGAGCACTTGTACGCAGGTTTC	TTTGTTCTGGCTTTCCACTTTT	190
**Recipient strain**			
16S rRNA	GCACAAGCGGTGGAGCAT	CTGGCAACAAAAGATAAGGGTT	199
ompA	TGAGCCTGGGTGTTTCCTAC	CAGAGCAGCCTGACCTTCC	167
ompF	GCAATGGCGACATGACCTA	CGTAATCGAAAGAACCAACGT	195
oxyR	AGTAACTGGTGGGTCTGTGCTT	CGTACCGTGCTGCGTGAG	194
rpoS	TGTTATCGCAGGGAGCCA	TTTTACCACCAGACGCAAGTT	179
lexA	GCAGGAAGAGGAAGAAGGGTT	CGGCTTGAATAAGGAAGGATC	115
recA	GTCGTTGACTCCGTGGCG	CGTGTTGGACTGCTTCAGGTTAC	138
luxS	GCGTGCCGAACAAAGAAG	CAGCCCATTGGCGAGATA	127
